# Protocol for investigating genetic determinants of posttraumatic stress disorder in women from the Nurses' Health Study II

**DOI:** 10.1186/1471-244X-9-29

**Published:** 2009-05-29

**Authors:** Karestan C Koenen, Immaculata DeVivo, Janet Rich-Edwards, Jordan W Smoller, Rosalind J Wright, Shaun M Purcell

**Affiliations:** 1 Department of Society, Human Development and Health, Harvard School of Public Health, Boston, MA 02115, USA; 2 Department of Epidemiology, Harvard School of Public Health, Boston, MA 02115; 3 Channing Laboratory, Brigham and Women's Hospital, Boston, MA 02115, USA; 4 Department of Psychiatry, Psychiatric and Neurodevelopment Genetics Unit, Center for Genetic Research Massachusetts General Hospital and Harvard Medical School, Boston MA 02114, USA; 5 The Broad Institute, Cambridge, MA 02141, USA

## Abstract

**Background:**

One in nine American women will meet criteria for the diagnosis of posttraumatic stress disorder (PTSD) in their lifetime. Although twin studies suggest genetic influences account for substantial variance in PTSD risk, little progress has been made in identifying variants in specific genes that influence liability to this common, debilitating disorder.

**Methods and design:**

We are using the unique resource of the Nurses Health Study II, a prospective epidemiologic cohort of 68,518 women, to conduct what promises to be the largest candidate gene association study of PTSD to date. The entire cohort will be screened for trauma exposure and PTSD; 3,000 women will be selected for PTSD diagnostic interviews based on the screening data. Our nested case-control study will genotype1000 women who developed PTSD following a history of trauma exposure; 1000 controls will be selected from women who experienced similar traumas but did not develop PTSD.

The primary aim of this study is to detect genetic variants that predict the development of PTSD following trauma. We posit inherited vulnerability to PTSD is mediated by genetic variation in three specific neurobiological systems whose alterations are implicated in PTSD etiology: the hypothalamic-pituitary-adrenal axis, the locus coeruleus/noradrenergic system, and the limbic-frontal neuro-circuitry of fear. The secondary, exploratory aim of this study is to dissect genetic influences on PTSD in the broader genetic and environmental context for the candidate genes that show significant association with PTSD in detection analyses. This will involve: conducting conditional tests to identify the causal genetic variant among multiple correlated signals; testing whether the effect of PTSD genetic risk variants is moderated by age of first trauma, trauma type, and trauma severity; and exploring gene-gene interactions using a novel gene-based statistical approach.

**Discussion:**

Identification of liability genes for PTSD would represent a major advance in understanding the pathophysiology of the disorder. Such understanding could advance the development of new pharmacological agents for PTSD treatment and prevention. Moreover, the addition of PTSD assessment data will make the NHSII cohort an unparalleled resource for future genetic studies of PTSD as well as provide the unique opportunity for the prospective examination of PTSD-disease associations.

## Background

Posttraumatic stress disorder (PTSD) occurs following exposure to a potentially traumatic life event and is defined by three symptom clusters: reexperiencing, avoidance and numbing, and arousal.[1] The majority of American women will be exposed to a traumatic event, although only a minority of such women will develop PTSD.[2,3] Still, the disorder is common: at least one in nine American women will meet criteria for the diagnosis in their lifetime.[3] Twin studies suggest genetic influences account for substantial proportion of the variance in PTSD risk among trauma exposed persons[4,5] but little progress has been made in identifying variants in specific genes that influence liability to PTSD. The few existing candidate gene studies in PTSD have been limited by methodological problems including convenience samples, focus on chronic rather than lifetime PTSD cases, inadequate power, poorly matched controls and the failure to assay all common variation in genes examined. This paper describes a protocol designed to identify genetic determinants of PTSD in women.

### Scope of the Public Health Problem

Posttraumatic stress disorder (PTSD) is common among American women with one in nine meeting criteria for the diagnosis at some point in their lives. Women who develop PTSD following trauma are at increased risk of major depression,[6] substance dependence,[7] impaired role functioning, and reduced life course opportunities, including unemployment and marital instability,[8] and health problems. [9-11] Women's lifetime risk of PTSD is twice that of men.[3] This sex difference is due to women's greater exposure and vulnerability to interpersonal violence.[2,3] Of all civilian traumas, interpersonal violence events are associated with the highest conditional risk of developing PTSD.[3,12,13] Women are both more likely than men to experience severe and repeated interpersonal violence throughout their lives and to develop PTSD following such experiences.[2,3,12,14,15] Thus, studies aimed at understanding the etiology of PTSD among women must comprehensively assess interpersonal violence exposure.

Only some women are vulnerable to the adverse effects of traumatic events. Only about half of female victims of even the most severe interpersonal violence such as a completed rape develop PTSD.[2,3,16] Two meta-analyses of PTSD risk factors have come to some consensus as to the key factors influencing PTSD vulnerability. These include small but consistent effects on risk for pre-trauma factors such as family psychiatric history, pre-trauma psychological adjustment, child abuse, other previous trauma exposures, and general childhood adversity.[17,18] Characteristics of the traumatic experience were found to be particularly important, especially trauma severity, perceived life threat and peri-traumatic emotional reactions such as dissociation.[17,18] A dose-response relation between severity of exposure and conditional risk of developing PTSD has been well-documented.[13,19] Post-trauma social support also appears to play a role.[17,18]However, the risk factors models supported by meta-analytic studies explain only about 20% of the variance in PTSD; clearly new variables need to be incorporated into models of PTSD vulnerability. Genetic factors, in particular, have been absent from most epidemiologic PTSD risk factor studies.

### PTSD is Heritable

As we [20-24] and others [25-27] have reviewed elsewhere, genetic factors are important in the etiology of PTSD. Family studies indicate that the prevalence of PTSD in relatives of PTSD probands is elevated as compared to relatives of individuals similarly trauma-exposed who did not develop PTSD. Cambodian refugee children whose both parents had PTSD were five times more likely to receive the diagnosis than children whose parents did not have PTSD.[28] Similarly, parents of children who developed PTSD in response to a serious injury were more likely to develop PTSD themselves.[29,30] Adult children of Holocaust survivors with PTSD had a higher risk of PTSD following trauma compared to adult children of Holocaust survivors without PTSD.[31,32] Likewise, twin studies have all shown elevated risk of PTSD in the monozygotic (MZ) co-twin of a PTSD proband relative to that seen in dizygotic (DZ) co-twins.[4,5,20] Data from twin studies indicate genetic influences account for about one-third of the variance in PTSD risk.[4,5]

### Methodological and Conceptual Limitations of PTSD Association Studies

The association method tests whether variation in a gene is correlated with an outcome (e.g. PTSD). This method detects genes of small effect and, until the recent development of genome-wide association studies (GWAS), had been the method of choice for molecular genetic studies of complex disorders. [33-36] However, to date, limited progress has been made in identifying variation in specific genes that increase risk for PTSD. The importance of genetic influences on PTSD risk have been recognized for half a century,[26] however, as of this writing, only 17 candidate gene studies of PTSD have been published. These are reviewed elsewhere [21].

#### Selection of Controls

The biggest challenge to PTSD candidate gene studies is appropriate control selection. According to epidemiologic principles,[37] controls should be selected from the same underlying population as the cases, representative of all controls with regard to exposure, and identical to the exposed cases except for the risk factor (in this case the genetic variant) under investigation. One practical implication of this last principle, referred to as "exchangeability" between cases and controls, is that controls must be similar to cases in severity of trauma exposure; several PTSD candidate gene studies do not report assessing trauma exposure in controls. [38-40] Violation of the exchangeability principle increases the likelihood that positive associations may be biased due to confounding factors and, in addition to the small sample sizes used in many studies, makes negative associations difficult to interpret. Our study addresses these limitations through proposing a large case-control study nested within a prospective longitudinal cohort where cases and controls will be matched on trauma exposure.

#### PTSD comorbidity

[3,41,42] A family history of psychiatric disorders is a consistent risk factor for developing PTSD.[17,18,42,43] Preexisting psychiatric disorders, particularly conduct disorder, major depression and nicotine dependence, also increase PTSD risk.[19,42,44-46] At the same time, PTSD increases risk for first onset major depression,[6] alcohol, drug, and nicotine dependence.[7,47] The incidence of other psychiatric disorders is not higher in individuals who experience trauma but do not develop PTSD. This fact has led to the suggestion that PTSD represents a generalized vulnerability to psychopathology following trauma.[42] This high PTSD comorbidity with other mental disorders raises the question of what to do about other disorders in genetic studies of PTSD.

Moreover, some of the genetic influences on PTSD overlap with those on other psychiatric disorders. [48-51] The extent of the overlap varies with the disorder studied. Data from the Vietnam Era Twin (VET) Registry suggests the largest overlap is with major depression; genetic influences common to major depression account for 57% of the genetic variance in PTSD.[52] Common genetic influences on major depression and PTSD is supported by molecular studies; the serotonin transporter promoter *s/s *polymorphism is implicated in both disorders.[38,53,54] Polymorphisms in FKBP5, a glucocorticoid-regulating cochaperone of stress proteins, which were associated with recurrence of major depressive episodes and response to antidepressant treatment[55] have also been associated with peri-traumatic dissociation,[56] a risk factor for PTSD and with PTSD symptoms among adults exposed to two or more types of child abuse[57]. Shared genetic influences explain part of the overlap between PTSD and alcohol and drug dependence,[50] panic disorder and generalized anxiety disorder,[49] and nicotine dependence.[46] This suggests some of the genes that influence risk for other mental disorders may also influence risk for PTSD. Moreover, the presence of other psychiatric disorders, particularly major depression, in trauma-exposed controls may attenuate the possibility of finding a positive PTSD-gene association. Our study addresses these issues by considering candidate genes for other psychiatric disorders (e.g. SLC6A4 for major depression) known to be comorbid with PTSD and by assessing major depression in trauma-exposed controls and conducting stratified analyses to test whether gene-PTSD associations are similar in cases with and without major depression.

#### Gene-environment interactions

PTSD is considered a 'complex' disorder in that there is likely no one gene or environmental factor that is sufficient for its development. Rather, there are likely many different genes, combined with many different trauma exposure and other environmental characteristics, which contribute in a probabilistic fashion to liability for developing PTSD in the general population.[58] Trauma timing, type, and severity may be important modifiers of genetic risk in PTSD as they have been shown to be important risk factors for PTSD in epidemiologic and meta-analytic studies.[12,13,17,18,45,59] Individuals whose first trauma occurs in childhood as opposed to adolescence or adulthood are at particularly high risk of developing the disorder.[13,17,18,60,61] Childhood abuse prospectively predicts trauma exposure in adolescence and adulthood; victims of childhood sexual abuse, in particular, are at increased risk of being raped later in life.[60] The conditional risk of developing PTSD is higher for interpersonal violence events, such as rape, than for other types of traumatic events (e.g. sudden unexpected death).[2,59,61]A dose-response relation between severity of exposure and conditional risk of developing PTSD has also been well-documented.[3,13,19] Severity of child maltreatment modified the association between MAOA genotype and antisocial behavior in European-American males [62-64]and the association between SLC6A4 genotype and depression in abused children.[65,66] A recent study demonstrated that severity of child abuse, but not adult trauma, modified the association between polymorphisms in FKBP5 and adult PTSD symptoms[57]. Thus, the data suggest age of first trauma predicts PTSD because younger individuals, particularly children, have fewer coping skills and resources to recover from the traumatic event. At the same time, more severe and/or repeated trauma exposure increases risk of PTSD because earlier stressors sensitize individuals to the effects of later stressors. We will consider whether timing, type, and severity of trauma exposure modify the association between genetic risk variants and PTSD.

### Candidate Genes Influencing PTSD Phenomenology

The diagnosis of PTSD requires that a person "experienced or witnessed, or was confronted with an event or events that involved actual or threatened death or serious injury, or a threat to the physical integrity of the self or others" (Criterion A1) and the person's response to the event involved "fear, helplessness or horror" (Criterion A2). Although many different types of experiences can meet these criteria, uncontrollable and threatening events such as rape, childhood abuse, and military combat are consistently associated with the highest conditional risk for developing PTSD.[3,12,59] Threatening events initiate the body's "fight-or-flight" response via the hypothalamic-pituitary-adrenal (HPA) axis and the locus coeruleus and noradrenergic system. These systems have important reciprocal interconnections with the amygdala and hippocampus, limbic structures involved in fear conditioning and memory consolidation, and with pre-frontal brain structures necessary for extinction of fear memories and reward motivation. Initially, this neurobiological stress response is considered adaptive; it mobilizes energy, increases vigilance and focus, facilities memory formation and depresses the immune response.[67] When the acute threat has passed, an elaborate negative feedback system will return the body to homeostasis. However, in some individuals this acute, adaptive response to threat becomes persistent and pathological.

The fear conditioning model for PTSD pathogenesis is most succinctly described by Pitman and Delahanty[68]: "A traumatic event (unconditioned stimulus) overstimulates endogenous stress hormones (unconditioned response); these mediate an overconsolidation of the event's memory trace; recall of the event in response to reminders (conditioned stimulus); releases further stress hormones (conditioned response); these cause further overconsolidation; and the overconsolidated memory generates PTSD symptoms. Noradrenergic hyperactivity in the basolateral amygdala is hypothesized to mediate this cycle."(p. 99). This persistent pathological response to uncontrollable stress is captured in the three symptom clusters of PTSD: (1) reexperiencing or reliving of the traumatic event; (2) avoidance of trauma reminders (which prevents extinction of the fear memory) and emotional numbing; and (3) generalized hyperarousal or hypervigilance. Although many individuals will experience some of these symptoms in the immediate days and weeks following a trauma, only a minority of individuals show the persistent symptoms required for the PTSD diagnosis. Moreover, the disorder will become chronic for almost 50% of those who meet diagnostic criteria.[8,69-71] The chronicity of PTSD reflects the persistence of conditioned fear memories. We posit inherited vulnerability to PTSD is mediated by genetic variation in three specific neurobiological systems whose alterations are implicated in enhanced fear conditioning: (1) HPA axis, (2) locus coeruleus and noradrenergic system, and (3) limbic-frontal neuro-circuitry of fear. The evidence supporting these genes has been reviewed in detail elsewhere[21].

### Specific Aims

We propose to use the unique resource of the Nurses Health Study II (NHSII), a prospective cohort of 68,518 women, to conduct what promises to be the largest candidate gene association study of PTSD to date. We will use a nested case-control study design to identify 1000 women who developed PTSD following trauma exposure and 1000 controls that experienced similar traumas but did not develop PTSD.

#### Primary Aim

Detecting genetic variation associated with risk for PTSD. The primary aim of this study is to detect variants of specific genes that predict the development of PTSD following trauma. We posit inherited vulnerability to PTSD is mediated by genetic variation in three specific neurobiological systems whose alterations are implicated in PTSD etiology:

A. Hypothalamic-pituitary-adrenal axis (e.g. CRH, CRH-R1, CRH-R2, CRH-BP, GCCR, GCR2, FKBP5)

B. Locus coeruleus/noradrenergic system (e.g. SLC6A2, DBH, COMT, ADRA2C, ADRB1&2, NPY, NPYR1&2)

C. Limbic-frontal neuro-circuitry of fear (e.g. BDNF, SLC6A3, DRD2, GRP, STMN1, OPRM1, SLC6A4, CREB1)

#### Secondary Aim

Dissecting genetic influences on PTSD in the broader genetic and environmental context. This secondary, exploratory aim will only be conducted for candidate genes that show significant association with PTSD in detection analyses. Specifically we will:

A. Conduct conditional tests to help identify the causal genetic variant among multiple correlated signals.

B. Test whether the effect of PTSD genetic risk variants is moderated by age of first trauma, trauma type, and trauma severity. We hypothesize that the effect of PTSD genetic risk variants will be magnified among women whose first trauma occurred in childhood (rather than adolescence or adulthood), among those exposed to interpersonal violence versus other traumatic stressors, and among those with more severe trauma exposure.

C. Explore gene-gene interactions using a novel gene-based statistical approach.

## Methods and design

### Cohort Establishment and Sampling Frame

The source population for this study will be participants in the ongoing prospective NHSII. In 1989, the NHSII cohort of 116,678 female registered nurses from the 14 most populous US states aged 24–44 in 1989 was established (PI, Walter Willett Grant NIH CA50385). The cohort has been followed by biennial mailed questionnaires inquiring about risk factors and incidence of disease mailed in June of odd-numbered years (1997, 1999, 2001, etc.). In 2001, the *2001 Violence Questionnaire *that was mailed to 91,297 NHSII participants (excluding only those who had previously requested short form questionnaires or who required more than four mailings before responding to the 1999 main questionnaire.) Non-respondents received a single reminder postcard. The 68,518 women who completed the *2001 Violence Questionnaire *(PI, Rosalind Wright Grant NIH XXXXX) comprise the sampling frame for this study. In 1997–99, plasma DNA samples were collected from a random sample of 29,613 participants, 25,021 of whom also answered the *2001 Violence Questionnaire*. Measures included in the *2001 Violence Questionnaire *are described in detail below.

### Ethical Approval

This research protocol has been approved by the Partners Human Research Committee (Protocol # P-002325/5) and is in compliance with the Helsinki Declaration.

### Stage 1: Supplemental Survey

**Figure 1:**
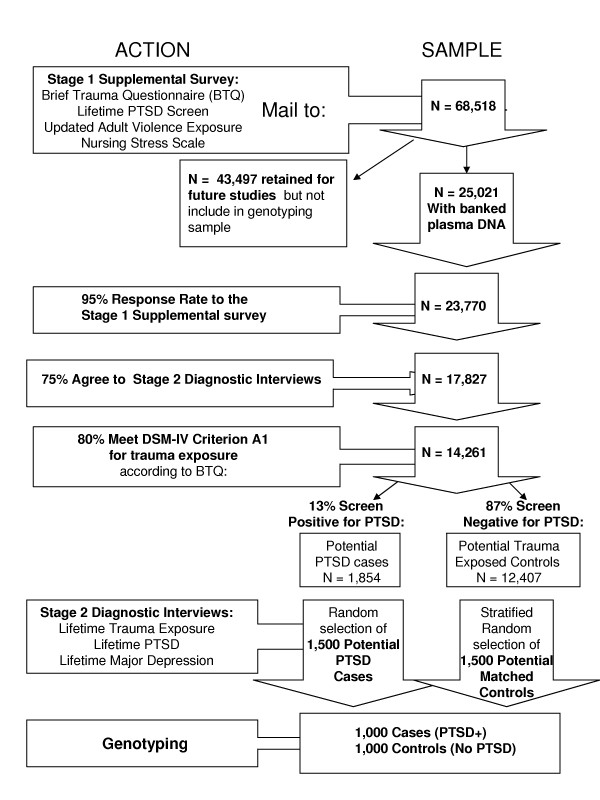
**Flowchart for case-control selection**.

Figure 1 provides a flow chart of the study design. In the first stage of the study, 68,518 women will be mailed the *2007 Supplemental Survey*. The survey will include the *Brief Trauma Questionnaire*, *Lifetime PTSD screen*, and updated adult violence exposure described in more detail below under *Measures*. The screening data will be used to efficiently sample cases and controls for the PTSD and major depression diagnostic interviews from the 25,021 women with banked plasma DNA. We are collecting screening data on all women because we will shortly have buccal DNA samples collected on 30,000+ additional women who answered the *2001 Violence Questionnaire*. The availability of survey data on all 68,518 women will make it possible to conduct future replication studies.

### Stage 2: Diagnostic Interviews for PTSD and Major Depression

The second stage will involve selecting potential cases and controls for diagnostic interviews. This will start with the 25,021 women who returned the *2001 Violence Questionnaire *and have banked DNA samples. Since these women have a 99% response rate on bi-annual questionnaires, we conservatively project that at least 95% will return the 2007 Supplemental Survey (n = 23,770) and that at least 75% of those who return the survey will agree to follow-up interviews. This gives us an estimate of 17,827 women from whom to select potential cases and controls for interviews. Our estimates of trauma exposure and PTSD prevalence in this sample are based on data from epidemiologic surveys using the DSM-IV criteria.[59] Thus, we estimate that at least 80% of the 17,827 women will meet DSM-IV Criterion A1, defined as exposure to at least one event that "involved actual or threatened death or serious injury, or a threat to the physical integrity of self or others," for trauma exposure (n = 14,261). Of these, 13% are projected to screen positive for PTSD (n = 1,854 potential cases) and the remaining are projected to screen negative (n = 12,407 potential controls). A total of 1,500 potential cases and 1,500 controls will then be selected for diagnostic interviews. Finally, 1,000 women with lifetime PTSD and 1,000 women with similar trauma who never met criteria for lifetime PTSD will be selected for genetic analyses.

### Integration of this project with the larger NHSII study

This study will take advantage of the resources of the ongoing NHSII study, whose core functions including the infrastructure of data collection and follow-up procedures, data management, and study oversight are funded by CA50385 (PI, Walter Willett, PI). Below we describe these core functions.

#### Data collection and follow-up procedures

Every two years (including 2005 and 2007), a follow-up questionnaire is mailed to all cohort members. These "main questionnaires" collect information on diet, physical activity, medication use, reproductive history, use of postmenopausal hormones, cigarette smoking, and incident disease (e.g. heart attacks). Up to six repeated mailings of the main questionnaire are sent to persistent non-respondents. Each year we are notified of more than 10,000 address changes and some mail is returned as undeliverable. Using a flow chart, these women are traced through direct contact with the local postmaster, State Boards of Nursing, credit bureau and web-based searches, former neighbors, and with contact persons designated by the study participant on past questionnaires. Through these approaches, only 350 women from the entire cohort remain as unforwardable. To maintain a high response rate, we continue to send certified mail to participants who do not respond after up to five mailings of the follow-up questionnaires. Through these mailing procedures we have achieved 98% response rate among women who returned the *2001 Violence Questionnaire *and 99% among women with banked DNA samples. Every four years, most recently 2005, we call non-respondents to the certified mailing to maximize follow-up and maintain contact. We have telephone numbers for over 62,000 of the study members and can access numbers for most of the rest of the cohort by sending a computer tape of names and addresses to the company Experian.

#### Data management

Questionnaire forms are printed using a high precision process to optimize the optical scanning of returned forms. The use of an optically scannable questionnaire reduces data entry errors to about 3 to 4 errors per 10,000 columns and provides substantial cost savings. Error rates are further reduced through verification routines. Returned questionnaires are counted daily and opened. Questionnaires are first visually examined to observe whether they were completed. For questions that have been inappropriately left blank, a "Pass Through" bubble is marked by the coder to indicate that this is an actual blank field. Completed forms are optically scanned using the NCS Pearson 5000 i scanner at the Channing Laboratory. Scanned data are passed through a verification program to check ranges of variables and consistency between responses (e.g., if a date of diagnosis was recorded was the disease itself reported?). All actual errors are checked against the paper copy and corrected online. This verification routine then writes a new data file representing the data from the batch of scanned questionnaires. The verification program is re-run on all batches that have passed through the program to catch any errors which have been overlooked. Once every questionnaire has been coded and scanned, all the data batches are merged together and sorted by ID to create a record of respondents to a questionnaire cycle. The ID will be used to link data from the *2001 Violence Questionnaire *and *2007 Supplemental Questionnaire *to data from the main questionnaires. The name and address file is maintained on a computer that is separate from the questionnaire data. This machine has special limited access, restricted to senior staff members to further protect the identity of respondents.

### Detailed Description of Phenotypic Measures and Data Collection Procedures

#### 2001 Violence Questionnaire

Briefly, measures were selected that have good validity and reliability[72] including: an abbreviated form of the *Childhood Trauma Questionnaire *(CTQ, a measure of emotional abuse and neglect until age 12), [73-75] an abbreviated version of the *Revised Conflict Tactics Scale*,[76] questions regarding inappropriate sexual touching or forced sex adapted from the *Sexual Experiences Survey*,[77,78] emotional abuse assessed with the *Women's Experience of Battering survey*, [79-81] and a series of questions regarding adult emotional, physical, and sexual abuse by an intimate partner adapted from the *McFarlane Abuse Assessment Screen*.[82] Questions on stalking from the *National Violence Against Women Survey*[83] were also included.

#### Stage 1 2007 Supplemental Survey

Supplemental survey data collection and management will be conducted according to the standard procedures used for the standard bi-annual surveys and is described in above. This will include up to three mailings of the questionnaire to non-responders.

##### Brief Trauma Questionnaire (BTQ)

The BTQ will be used to determine whether a woman meets Criterion A1 for traumatic exposure according to the DSM-IV PTSD diagnosis. It is a brief self-report questionnaire designed to assess 10 traumatic events including physical assault, car accidents, natural disasters, and unwanted sexual contact. It is derived from the *Brief Trauma Interview*.[84,85] Interrater reliability kappa coefficients for the presence of trauma that met Criterion A1 for trauma exposure according to the DSM-IV were above .70 (range .74–1.00) for all events except illness (.60). Criterion validity of the BTQ is supported by strong association with acute trauma response as measured by dissociation.[86]

##### Lifetime PTSD screen (L-PTSD screen)

The *L-PTSD screen *will be used to identify potential PTSD cases and controls among woman who meet Criterion A1 for traumatic exposure according to the BTQ. The screen is adapted from Breslau et al.'s 7-item screening scale for DSM-IV PTSD.[87] The scale queries 5 avoidance symptoms and 2 arousal symptoms. Endorsement of 4 or more symptoms in relation to the worst trauma has been shown to classify PTSD cases with a sensitivity of 85%, specificity of 93%, positive predictive value of 68%, and negative predictive value of 98%. The cutoff point is optimized for two-stage designs such as that used in this study where the first phase is designed to maximize the number of true cases of PTSD and the second phase is expected to reclassify those who were wrongly classified as having the disorder. For the purposes of this study, participants will be asked to identify their worst event on the BTQ and determine whether they have experienced the symptoms in relation to that trauma.

##### Adult Violence Exposure Update

The *2007 Supplemental Questionnaire *will also be used to provide an update on adult violence exposure occurring since 2001. The update will include a series of questions regarding whether participants had experienced adult emotional, physical, and sexual abuse by an intimate partner since 2001; these questions were adapted from the *McFarlane Abuse Assessment Screen*.[82] Information on emotional abuse since 2001 will be assessed with the *Women's Experience of Battering *survey, a valid and reliable 10-item scale which assesses the woman's perceptions of fear, autonomy vs. control of her life by an intimate partner.[80] Questions on stalking from the *National Violence Against Women Survey*[83] will also be included.

#### Stage 2 Diagnostic Interviews

##### Participation in diagnostic interviews

Women will also be asked as to whether they would be willing to participate in a phone interview about their life experiences and reactions to those experiences. Women who agree to participate will also be asked to indicate the best phone number, email address and days/times of the week they would prefer to be contacted. For cost efficiency, the effect of genotype on risk of PTSD will be examined using a nested case-control design. The second stage of this study will involve selecting 1,500 potential cases and 1,500 controls for diagnostic interviews.

Potential cases will be defined as women who: 1) meet Criterion A1 for trauma exposure according to the BTQ and 2) endorsed four or more symptoms on the L-PTSD screen. Of the projected 1,854 cases, 1,500 will be randomly selected for diagnostic interviews. Once cases are selected, we will stratify them based on current age (42–51, 52–62), ethnicity, and trauma-exposure severity. Trauma-exposure severity will be operationalized using data from the BTQ, *2001 Violence Questionnaire*, and updated adult violence exposure. Following the strategy of Breslau,[2,88] Stein,[5] and Resnick,[14] traumatic events will be classified as either interpersonal violence events (IPV) or other traumatic stressors (OTS). For the purpose of stratification, therefore, trauma severity will be classified as: 1) low for women who have only experienced an OTS and no IPV events; 2) medium for women who have experienced at least one IPV event; 3) high for women who have experienced two IPV events; and 4) highest for women who have experienced more than two IPV events. Our classification of trauma severity is based on two well-established epidemiologic findings. First, conditional risk for PTSD in women is highest for IPV events. Second, exposure to multiple traumas, particularly IPV events, increases the conditional risk of developing PTSD.[2,3,59-61,88]

Potential controls will be matched to cases on trauma-exposure severity; controls are women who were exposed to similar traumatic events as cases but did not develop PTSD as of the date they filled out the *2007 Supplemental Questionnaire*. Controls must minimally meet the following criteria: 1) have been exposed to a traumatic event that meets the DSM-IV A1 criterion according to the BTQ and 2) endorse less than four symptoms on the L-PTSD screen. We project that 12,407 women will meet those criteria. For matching, we will stratify controls based on age (42–51, 52–62), ethnicity, and trauma-exposure severity and then randomly select 1500 controls within strata so that the distribution of strata for our controls *matches *that for cases. Given our large number of potential controls, we will be able to make a strong match. We will also consider restricting selection to those who meet trauma-exposure criteria but have low (< = 2) L-PTSD screen scores.

#### Diagnostic Interviews

For women who consent to be interviewed and meet the above criteria for case-control selection, contact information, including telephone numbers and home addresses, for 1,500 potential cases and 1,500 potential controls will be forwarded to Shulman, Ronca, & Buvucalas Incorporated (SRBI). Women selected for interviews will be notified via postcard.

##### Sample Tracking and Location

Women who have agreed to be interviewed will also have provided updated phone numbers. If women agree to be interviewed but omit phone numbers from their survey, we have telephone numbers for over 62,000 of the study members and can access numbers for most of the rest of the cohort by sending a computer tape of names and addresses to Experian. Every effort will be made to present SRBI with fully updated names and contact information for all potential interviewees.

##### Survey Interview Procedures

Procedures that SRBI will use to contact interviewees are as follows. All phone numbers are produced on a location sheet and sent to specially trained locators who will attempt every phone number up to 20 times and use a custom script to help ascertain if the respondent is at that number. If a respondent is identified with the same name and SSN, they are asked if an interviewer could call them back to speak with them about the project. If a new phone for the respondent is identified, it is added to the tracking sheet and dialed. If a new address or city is found, locators call directory assistance to get the number. Every telephone number obtained will be attempted, and each working number will be screened by our locators for location. If the telephone number does not yield the correct respondent, locators will first confirm that they have dialed the correct number. They will ask if anyone by the respondent's name has ever lived there, if they know anyone by that name and how to get in touch with the respondent. If someone at that number has the same name as the respondent, locators will confirm that they are speaking with the correct person. Once the interviewee has been located and consent for call-back obtained, their name will be given to a trained interview. The interviewer assigned to conduct the diagnostic interview will call back 50 times or more if necessary to obtain the projected response rate within the field period.

*Lifetime trauma exposure and PTSD *will be assessed following the procedures used by Breslau in her epidemiologic studies of PTSD.[12,13,59,89]. The interview begins with an enumeration of traumatic events operationalized by Criterion A1 and A2 (response to trauma "involved intense fear, helplessness, or horror") of the DSM-IV definition for PTSD. An endorsement of an event type is followed by questions on the number of times an event of that type had occurred and the respondents' age at each time. A procedure was implemented for identifying complex, interrelated events (e.g. a subject was raped, beaten-up, and threatened with a weapon on the same occasion) and codes them as a single distinct event. The respondent is then asked to identify her worst event. PTSD is evaluated in relation to the worst event using a slightly modified version of the Diagnostic Interview Schedule-IV (DIS-IV[90] and the Composite International Diagnostic Interview (CIDI) Version 2.1.[91] The instrument is a fully structured diagnostic interview designed to be administered by experienced interviewers without clinical training. Subjects' responses are used to diagnose DSM-IV PTSD. A validation study conducted by Dr. Breslau[92] found high agreement between the telephone interview and independent clinical re-interviews conducted on the telephone by two clinicians blind to respondents initial PTSD diagnosis (sensitivity = 95.6%, specificity = 71.0%). Research supports the validity of telephone as compared to face-to-face interviews for PTSD.[93]

*Lifetime Major Depression *will be assessed via the Composite International Diagnostic Interview (CIDI) Version 2.1[91] a structured instrument for use by trained lay interviewers. Diagnoses are based on *DSM-IV *criteria. Organic exclusions and diagnostic hierarchy rules are both applied in making diagnoses. Acceptable-to-good concordance between the CIDI diagnoses and blind clinical diagnoses has been shown.[94] Research supports the validity of telephone as compared to face-to-face interviews for major depression.[95,96]

##### Quality control and reliability of interview data

We will maximize the quality of interview data by using a computer-assisted telephone interview (CATI) procedure in which each question in the highly structured diagnostic interview appears on a computer screen and is read verbatim to respondents. Use of CATI incorporates complex skip patterns into the interview, eliminates post-interview coding errors, and reduces interviewer's inadvertent failure to ask some interview questions. Supervisors listening to real-time telephone interviews while monitoring the CATI interview on their own computer perform random checks of each interviewer's assessment behavior and data entry accuracy at least twice per shift. When an error is detected, supervisors require its correction and discuss it with the interviewer after the interview. If the error is detected again in following interviewers, the interviewer is removed from the study. Use of highly structured CATI interviews with well-trained carefully monitored interviewers provides excellent quality control during data collection and data entry processes. The CATI format has been used by SRBI in many epidemiologic studies of PTSD including the National Women's Study,[14] World Trade Center Disaster Study,[97] 2004 Florida Hurricanes study,[98] and the National Violence Against Women Survey.[83,99]

#### Case-control Selection for Genotyping

Our simulations (see below) indicated that samples of 1,000 cases and 1,000 controls would provide good power to test our primary detection hypotheses. Thus, 1,000 cases will be randomly selected from among the interviewed women who receive a PTSD diagnosis and 1,000 controls will be randomly selected from among the interviewed women who report exposure to a traumatic event and who do not meet diagnostic criteria for lifetime PTSD.

#### Laboratory Methods and Genotyping

##### Biosample Collection

Blood collection kits were sent to a random sample of ~30,000 participants who indicated on their 1997 NHSII questionnaire that they would be willing to send us a blood sample. Each participant arranged for the blood sample to be drawn. The blood samples were returned to via overnight courier. We collected blood samples for 25,021 women who also completed the *2001 Violence Questionnaire*.

##### Sample processing

After arrival in the lab, blood samples were centrifuged and aliquotted into cryotubes as plasma, buffy coat, and red blood cells. Cryotubes are stored in liquid nitrogen freezers at a temperature of -130°C. Freezers are alarmed and continuously monitored; no samples have inadvertently thawed. Buccal cell samples are processed using ReturPureGene DNA Isolation Kit (Gentra Systems, Minneapolis, MN) to extract genomic DNA from human cheek cells. Buccal samples are logged in on receipt, the DNA is extracted, and the extracted DNA is archived in liquid nitrogen freezers using specific tracking software. The average DNA recovery from these specimens as measured by PicoGreen is 59 ng/ul.

##### DNA extraction in 96-well format

We can extract high-quality DNA from buffy coats from 96 samples in 4–5 hours. 50 μl of buffy coat are diluted with 150 μl of PBS and processed via the QIAmp (Qiagen Inc., Chatsworth, CA) 96-spin blood protocol. The protocol entails adding protease, the sample, and lysis buffer to 96-well plates. The plates are then mixed and incubated at 70°C, before adding ethanol and transferring the samples to columned plates. The columned plates are then centrifuged and washed with buffer. Adding elution buffer and centrifuging elutes the DNA. The DNA concentrations are calculated in 96-well format using a Molecular Dynamics spectrophotometer. The average yield from 50 μl of buffy coat (based on >1000 samples) is 5.5 μg with a standard deviation of 2.2 (range 2.2–16.4).

#### DNA genotyping methods

##### Genotyping

SNP genotyping will be performed at the Harvard Cancer Center Genotyping Core, a unit of the Harvard-Partners Genotyping Facility. The ABI Taqman system using a model 7900 detection device will be used for SNP allelic discrimination. This instrument uses probes with two dyes on opposite ends of a target sequence oligonucleotide to recognize SNP polymorphisms. One dye is a reporter dye, the other a quencher. When the probe is intact, the quencher suppresses fluorescence from the reporter; when the quencher and reporter are separated, the reporter emits a fluorescence signal. When the probe hybridizes exactly to its complement, the 5' exonuclease activity of Taq polymerase cleaves the probe and allows the signal to be detected. The Taqman system uses two probes to detect a SNP, one complementary to each allele. An advantage of the Taqman system is that ABI offers detection reagents for many polymorphic systems pre-synthesized and tested, "on demand." Detection reagents for other variants are ordered "on demand" through a user-friendly WWW interface.

We will use tag SNPs as an efficient way to assay common genetic variation. For example, the GCCR gene spans ~125 kb and contains 59 common SNPs in the most recent version of the HapMap. By selecting tag SNPs, (e.g. de Bakker *et al*[100]) based on the linkage disequilibrium profile across this gene in Caucasians, only 14 SNPs are needed to assay the common genetic variation (minor allele frequency > 0.10) with r^2^>0.8. In total, 16 tests are specified. The mean r^2 ^between typed and untyped variants is 0.96.

High density SNP mapping can indirectly assay other forms of common genetic polymorphisms, such as repeat length polymorphisms and insertions/deletions. With a sufficiently dense SNP panel, the vast majority of common variation (whether the variation is a SNP or not) will be assayed either directly or indirectly (via linkage disequilibrium, LD). For example, a specific repeat length polymorphism would have arisen on a particular chromosomal background. A dense SNP panel will be informative about the haplotype on which the repeat polymorphism arose, thereby providing a proxy for the repeat. Similarly, it is sometimes cited as a failing of SNP mapping that an association may be "only" due to LD as opposed to the true causal variant, which is often described in terms of epidemiological confounding. In contrast, it is precisely this "confounding" that makes SNP mapping feasible as a powerful and efficient way to scan common genetic variation without explicitly testing every single variant. Furthermore, unlike most confounding in classical epidemiology, confounding due to LD implies that the true variant must (in a homogeneous sample) be *physically proximal *to the correlated SNP which is vital in the goal of localizing the true signal. In any case, once an investigator has isolated an association signal to, say, several SNPs in a particular gene, there are other statistical methods that can identify if one or more markers are more likely than others to be the causal variant.

##### Selection of polymorphisms

We will use the most recently published HAPMAP[101] data to capture all common known variation (>1%) in the selected genes and conduct haplotype-based association tests. We will select SNPs for fine mapping using databases such as: dbSNP , HAPMAP , USC Genome Browser , and SNPselector . If genes are not included in the HAPMAP (e.g. CRH, STMN1, ADR2C, GCR2 [GRLL1]), we will use fine-mapping techniques to identify haplotypes in our sample.

##### Ancestry-informative marker set methods

Two different sets of markers will be used to assess for population stratification. First, we will use the AmpFLSTR Identifiler PCR Amplification Kit (Applied Biosystems (ABI), Foster City, CA), which provides data from a set of 16 loci useful for forensics purposes (D8S1179, D21S11, D7S820, CSF1PO, D3S1358, TH01, D13S317, D16S539, D2S1338, D19S443, vWA, TPOX, D18S51, D5S818, FGA, and amelogenin). The markers in this set are all co-amplified in a single PCR reaction. Second, we selected 21 markers known to have high *δ *between European Americans and African Americans, and in some cases Hispanic and Asian populations.[102] This marker panel includes markers D1S196, D1S2628, D2S162, D2S319, D5S407, D5S410, D6S1610, D7S640, D7S657, D8S272, D8S1827, D9S175, D10S197, D10S1786, D11S935, D12S352, D14S68, D15S1002, D16S3017, D17S799, and D22S274.

##### Genotyping quality control (QC) procedures

For all nested case-control study sets, we routinely add approximately 10% of repeated quality control samples as blinded specimens. These DNA samples are randomly nested in the sample sets with coded IDs that keep laboratory personnel blinded to QC status at all stages of the genotyping procedures. After genotyping is completed, but before any statistical analysis is performed, QCs are reviewed by a programmer. If errors are found, we seek to diagnose the source of the error. The very few errors that have occurred were mostly clerical errors in labeling scatterplots. If the source of the errors cannot be found, we would repeat all genotypes from the set where the error occurs.

### Statistical Analysis

#### Definitions of Key Variables

##### Lifetime PTSD and major depression

The diagnoses of PTSD and major depression will be made via diagnostic interview according the DSM-IV diagnostic criteria using a computer algorithm.

##### Trauma exposure

Timing, type and severity of trauma exposure will be determined via the diagnostic interview. Timing will be defined by age at first trauma (childhood < age 13). Type will be classified as either interpersonal violence (IPV; e.g. rape, sexual abuse, physical assault, domestic violence) or other traumatic stressors (OTS; car accidents, natural disaster) from the PTSD diagnostic interview. Trauma-exposure severity in cases will be defined by the type and number of events occurring before the onset of the first PTSD. Trauma-exposure severity in controls will be assessed as lifetime. Severity will be classified as high (2+ stressors) or low (1 stressor). This grouping may be adjusted depending on the distribution of exposures.

##### Potential confounders

Variables to be considered as confounders are those that may be common prior causes of exposures and outcomes. Under this definition, there are few potential confounds of the association between genetic variants and PTSD beyond population stratification. However, confounding is a concern for dissection analyses of trauma-gene interactions and PTSD. Many variables (e.g. childhood socio-economic status [SES]) could be considered common prior causes of the trauma-exposure severity-PTSD association. Through our diagnostic interviews, we will have age of onset for trauma exposure and PTSD. This will enable us to establish temporal relations between potential confounders, trauma exposure and PTSD diagnosis. Factors such as childhood SES that temporally precede trauma-exposure and PTSD and may be common prior causes of an observed association will be controlled for in the dissection analyses. A strength of the NHSII dataset is the array of prospective data on potential confounders that can be adjusted for in statistical analyses. The variable list is online: .

#### Statistical Analyses and Power for Primary Aim: Detection Stage

The primary aim of this study is to detect variants of specific genes that predict the development of PTSD following trauma. We hypothesize inherited vulnerability to PTSD is mediated by genetic variation in three specific neurobiological systems (HPA axis, locus coeruleus/noradrenergic system, limbic-frontal neuro-circuitry of fear) whose alterations are implicated in PTSD etiology.

Basic association analysis will be performed using the *PLINK*[103] and *Haploview*[104] software packages. The initial step of analysis is to perform a rigorous quality control procedure: high missing genotype rates (both per individual and per SNP) and deviations from Hardy-Weinberg genotype proportions are indicative of problems. Individuals and/or SNPs will be removed as needed, as will very rare and monomorphic SNPs. The basic association test assumes an additive effect of SNP genotype (for dichotomous traits, additivity is on the log-odds scale) and is a regression of the phenotype on genotype. It is also possible to assume dominant and recessive gene action, and to perform likelihood ratio tests comparing these three models to a more general 2 degree of freedom model. We propose to take account of potential covariates (such as subpopulation membership in the case of population stratification) either by directly incorporating covariates in the model, or by use of a permutation-framework (i.e. permuting phenotype labels only within subpopulations).

Information across multiple SNPs within a gene can be combined in two ways: via haplotype-based tests and gene-based tests. The *PLINK package *uses a weighted likelihood mixture of regressions model, to account for the potential ambiguity in statistically-inferred haplotypes, following the model of Schaid *et al*.[105] The posterior probabilities for each particular pair of haplotypes for each individual are calculated via the E-M algorithm; these posterior probabilities (of haplotype pair conditional on multilocus SNP genotype data) are used to weight the haplotype-PTSD association analysis. For *H *haplotypes, either a *H-1 *df omnibus test (looking for a joint effect of all haplotypes) or a series of *H *haplotype-specific tests, each with 1 df, can be conducted. The tests are likelihood ratio test statistics; both asymptotic and empirical significance values are available, as well as confidence intervals on parameter estimates. Based on the LD profile of each gene, haplotypes will either be formed across the entire gene, or restricted to regions of high LD/low haplotype diversity, e.g. using a haplotype block definition rule as implemented in the Haploview package[104]. In contrast, for *S *SNPs, a gene-based analysis simply considers the *S *cumulative sums of rank-ordered single SNP association chi-squared statistics (S_1_, S_1_+S_2_, S_1_+S_2_+S_3_, ...) and evaluates significance via permutation, which also corrects for having tested *S *different ranked sum scores. This method is a gene-based implementation of Ott & Hoh's[106] method that utilizes sum-statistics. A gene-based test might potentially be more sensitive to genes with multiple, less common variants having individually small effects on the phenotype.

Table 1 presents power calculations for our study. The sample is well powered to perform a comprehensive screening of common variation in ~30 genes. We used the Genetic Power Calculator[107] online resource to calculate power, assuming either that the causal variant (CV) is directly typed (an upper bound on power) or is in incomplete linkage disequilibrium (LD) (r^2 ^= 0.8) with a typed marker (effectively a lower bound, as the tag SNP selection is designed to capture all common variants with an r^2 ^of at least 0.8). The calculations below are based on 1000 cases and 1000 controls, assuming a prevalence of PTSD of 13%, a multiplicative true mode of gene action and that the test is a 1 df test allelic or haplotypic test. The calculations are parameterized in terms of a liability-threshold model: the CV explains either 1 or 2% of variation in a continuous, unobserved normally-distributed liability; the threshold is chosen to correspond to the known population prevalence of PTSD. That is, rather than a table ordered by fixed risk ratio (which would show that rare, low-penetrance alleles are undetectable by any practical study design given the sample-size requirements and that common, higher penetrance alleles are detected easily), we fix the variance explained in the table to restrict the presentation to the lowest range of effects likely to be achievable – and find that indeed genetic risk factors that explain considerably less than 2% of the variance in liability will be detected by our study. The implied genotypic relative risks (GRR) for the having one (het) or two (hom) copies of the risk allele relative to the baseline genotype are shown in Table 1.

**Table 1:** Power calculations for genetic study of 1,000 cases and 1,000 controls

			**Power (alpha = 0.05/12)**		**Power (alpha = 0.05/360)**	
Risk allele freq.	GRR(het)	GRR(hom)	Lower bound (r^2 ^= 0.8)	Upper bound (r^2 ^= 1)	Lower bound (r^2 ^= 0.8)	Upper bound (r^2 ^= 1)
**1% of variance in liability**						
0.010	2.65	4.86	0.87	0.95	0.58	0.75
0.025	1.95	3.25	0.87	0.94	0.57	0.74
0.050	1.65	2.49	0.86	0.94	0.55	0.72
0.100	1.45	2.02	0.84	0.93	0.53	0.70
0.250	1.31	1.67	0.83	0.92	0.50	0.67
0.500	1.27	1.59	0.81	0.91	0.47	0.65
0.750	1.34	1.75	0.79	0.89	0.45	0.62
0.900	1.56	2.33	0.76	0.87	0.40	0.57
**2% of variance in liability**						
0.010	3.59	6.55	1.00	1.00	0.96	0.99
0.025	2.48	4.54	1.00	1.00	0.97	0.99
0.050	1.99	3.37	1.00	1.00	0.97	0.99
0.100	1.69	2.61	1.00	1.00	0.96	0.99
0.250	1.47	2.05	0.99	1.00	0.95	0.99
0.500	1.42	1.94	0.99	1.00	0.93	0.98
0.750	1.53	2.25	0.99	1.00	0.91	0.97
0.900	1.96	3.50	0.98	1.00	0.87	0.96

To address the issue of multiple testing: power is given for three levels of type I error rate: 0.05, 0.05/12 (4.17e-3) and 0.05/360 based on 30 genes (1.39e-4) which correspond to a (conservative) control of family-wise error rate at the level of SNP (i.e. single test), gene and experiment respectively. In practice, during analysis we will use a less conservative permutation-based procedure to control for multiple testing: the conservative Bonferroni assumption is used only to facilitate power calculation. The "lower bound" on power is based on the reasonable assumption that tag SNP selection will improve efficiency (i.e. r^2 ^> = 0.8). We performed a set of coalescent simulations to determine the expected maximum r^2 ^that would result from completely random selection of SNPs, to provide an even more stringent lower bound on power. Using the CoSi simulator, we generated 50 kb haplotypes (i.e. corresponding to a typically-sized gene) with SNP frequency and LD profiles similar to those observed in Caucasian samples (assuming uniform mutation and recombination rates). We randomly designated one variant (minor allele frequency, MAF > 0.02) as the "CV" and then selected 12 variants (MAF > 0.02) as the typed SNPs. Across 500 replicates, the average maximum r^2 ^between a typed SNP and the (possibly typed but most likely unobserved) CV varied depending on the allele frequency of the CV, from approximately 0.5 for less common SNPs (MAF < 0.1) to 0.7 for more common CVs. Even in the scenario that the CV is rare and the tag SNP selection performs no better than chance, the expected marker density should ensure reasonable to good coverage of common variation. Power is still good under most circumstances: for a 1% CV with MAF of 0.1, the "lower bound" drops from 0.84 (r^2 ^= 0.8) to 0.78 for r^2 ^= 0.7 (0.58 for r^2 ^= 0.5), although experiment-wide power is poor in this case however, at 0.43 for r^2 ^= 0.7 (0.23 for r^2 ^= 0.5). For CVs explaining 2% of the variation in liability, power at the gene-wide level is still greater than 0.90 in almost all cases; experiment-wide power approximately ranges between 0.80 and 0.90 for r^2 ^= 0.7 (0.60 and 0.70 for r^2 ^= 0.5). In summary, even under the unlikely assumption that tag SNP selection adds no value whatsoever, and the conservative correction for all 360 single SNP tests assuming independence, the study is still adequately-powered at the experiment-wide level for multiple tests.

##### Approach to multiple testing

As well as limiting the number of tests performed via specific hypotheses, we propose to use a permutation-based framework to control for multiple testing. Within a gene, we will control the family-wise type I error rate at 5%: case labels are randomly permuted (possibly within subgroups to control for potential confounders) against all genotypes – this procedure maintains the correlational structure of the tests under all permuted replicates, and so is not conservative as the Bonferroni correction which assumes tests are independent. By comparing each observed test statistic within a gene against the *maximum permuted test statistic per replicate*, the empirical p-value will naturally control for multiple testing. (A similar logic can be applied to multiple, potentially correlated, phenotypes also.) At the gene-based level of analysis, controlling for the chance of *at least one false positive *is appropriate, as we will conclude a significant *gene*-disease association if at least one test within the gene is significant after correction. In contrast, we may wish to use a less stringent control across genes, to obtain an experiment-wide error significance value: here false discovery rate (FDR) procedures, that control the probability that a significant result is also a true one, may be more appropriate. We should note that, along with many areas in statistical genetics, this area is currently the subject of much methodological development and debate: as such, when the time comes to perform the analysis, the literature will be reviewed to formalize a specific analytic plan. Ultimately, replication in an independent sample will also be important to establish true associations.

##### Genetic overlap between PTSD and major depression

Based on epidemiologic studies, we estimate that the prevalence of lifetime major depression (MD) will be ~40% in PTSD cases and ~20% in trauma-exposed controls who never developed PTSD.[3,6,8,12,41,42]Given our sample of 1000 cases and 1000 controls, this will be the first PTSD candidate gene study to date with adequate numbers of PTSD cases with and without MD to conduct exploratory analyses examining the complex relation between these two disorders in trauma-exposed women. To empirically address this potentially complex genetic relationship, for PTSD-associated variants we will, a) test whether genotype distribution differs within PTSD cases with and without MD, b) establish whether the association with PTSD holds after controlling for MD. These tests will be performed using PLINK: the first analysis is a standard association analysis performed in the PTSD case subsample; the second analysis will use the Cochran-Mantel-Haenzsel test for association in stratified tables (stratifying by MD status). In this way, we can ask whether the association is similar for PTSD cases with and without PTSD or is specific to PTSD, or to the PTSD+MD comorbid phenotype. Given genetic influences on MD explain 57% of the genetic variance in PTSD,[52] we predict most gene-PTSD associations to be similar for PTSD cases with and without MD.[108]

#### Statistical Analyses for the Secondary Aim: Dissection Stage

The goal of the *Detection *stage is to screen all genes for association using a simple, powerful and rigorous analytic approach. In this second *Dissection *stage, we propose a more detailed examination of any genes that pass the first stage, both to refine the association signal and to explore it in its broader phenotypic, genetic and environmental context. In particular, we consider: 1) conditional tests to determine the causal variant among multiple correlated association signals; 2) analysis of trauma timing, type, and severity; 3) a gene-based approach to detecting epistasis.

We will capitalize on the large sample size and conduct conditional analyses to help fine-map the causal variant within a region showing multiple significant associations. The use of haplotype information can, to some extent, help to determine whether specific associated SNPs and/or haplotypes are more likely to be only indirectly associated (via LD) as opposed to being the causal variant. The *PLINK *package enables a flexible specification of nested hypotheses which allows tests to be constructed that ask questions such as: *can this sole SNP or haplotype explain the entire association signal at a locus? does SNP A have an effect independent of SNP B or haplotype C? *For example, for two SNPs, alleles *A *and *B *(as opposed to alleles *a *and *b*) may both be associated with PTSD as well as with each other (due to LD). The basic analysis would not inform us as to whether both *A *and *B *are contributing independently to risk for PTSD, however. A conditional analysis might proceed as follows: if, for example, three haplotypes are observed, *AB*, *ab*, and *Ab*, then we can test each SNP controlling for the other, e.g. for the *A *allele the test is [*Ab *versus *ab *] and for the *B *allele it is [*AB *versus *Ab*]. The *PLINK *package (developed by Dr. Purcell) enables flexible specification of such conditional tests, for any number of SNPs and haplotypes. For example, testing the effect of *A *conditional on two other SNPs might entail fitting a model that equates the following haplotypes: [*ABC = aBC; AbC = abC; Abc = abc *] and comparing the fit (via likelihood ratio test statistic) with the full model which does not impose these equality constraints. The above model can be easily specified in *PLINK*. In summary, given a strong initial association signal, these analyses can help to determine which variants are causal and which are only indirectly associated. This analysis can never *prove *that a variant is causal: it can however, indicate which of a set of associated variants do not show simple independent causal effects and inform functional studies.

We will test whether trauma timing, type, and severity modify the association between genetic risk variants and PTSD. For genetic variants that pass the *Detection *stage (p < .05 after correction for multiple testing), we will perform a focused set of analyses that test for heterogeneity in terms of the timing, type, and severity of the trauma. We hypothesize that the effect of PTSD genetic risk variants will be magnified among women whose first trauma occurred in childhood (rather than adolescence or adulthood), among those exposed to interpersonal violence versus other traumas, and among those with more severe (high versus low) trauma exposure. Heterogeneity analyses can be performed using *PLINK*, which allows allelic and haplotypic coefficient to vary as a linear function of a measured covariate, e.g. instead of simply *g *the coefficient is estimated as *(g+bM*_*i*_*) *where *M*_*i *_is the measured covariate for individual *i*. A likelihood ratio test is constructed by comparison against the nested submodel with fixes *b *to 0, which indicates whether the association depends on the covariate. For any environmental measures coded as multiple categories, we shall use the Breslow-Day test of homogeneous odds ratios as implemented in *PLINK*.

Our sample of 1000 cases and 1000 controls is well powered to perform gene-trauma interaction analyses for genes associated with PTSD in detection analyses. To evaluate the statistical power to detect an interaction between genotype and trauma-exposure characteristics, we conducted a series of simulations considering a range of scenarios. Power was calculated as the proportion of simulated samples (out of 1000) that were significant for an alpha level = .01. The power to detect an interaction will depend on the minor allele frequency, prevalence of the high-risk trauma-exposure characteristic (e.g. childhood trauma versus later, IPV versus OTS, high versus low exposure severity), and the effect size for the interaction. We chose minor allele frequencies of .10, .25, and .50 to be comparable to minor allele frequencies of variants included in our study, e.g. the *s *allele of SLC6A4 has ~50% frequency in Caucasian populations. In all cases, we assumed a main effect of exposure, an allelic effect only in the high-risk exposure group, and alpha = .01. In summary, if the prevalence of the high-risk trauma-exposure characteristic was .10 or .25, power to detect interaction ranged from .80 to ~1.00 for a minor allele frequency of .10 or greater and an interaction RR of 1.5 or greater. If the prevalence of the high-risk trauma-exposure characteristic was .50, power was >.90–1.00 for a minor allele frequency of .10 or greater and interaction RR of 1.5 or greater.

As a more exploratory, secondary goal, we plan to evaluate evidence for epistatic gene-gene interaction, using a novel method which considers all SNPs in a pair of genes simultaneously in the test for interaction. The method has been validated both in simulation studies and via application to several datasets, e.g. detecting interaction between dysbindin and the BLOC-1 genes in schizophrenia, Morris et al.[109] The method, based on canonical correlation analysis, can be applied either as a case-only test for epistasis (more powerful but applicable only to unlinked genes and makes a more stringent assumption regarding population homogeneity) or the more traditional case-control approach. Comparing this approach to the standard pairwise SNP-by-SNP approach (e.g. Marchini et al.[110]) simulations have shown the increase in power. For example, using a dominant/complementary model of epistatic gene action, we simulated 5 genes (of which only two interacted) each with 10 SNPs, which leads to 250 SNP-by-SNP tests but only 25 gene-by-gene tests. We used permutation to generate empirical p-values and control for multiple testing: power is presented correcting for all tests in a particular gene-by-gene comparison, and also at the experiment-wide level. The standard SNP-by-SNP approach yields powers of 24% and 6% respectively, whereas the new approach gave 70% and 58% power. This novel approach is therefore considerably more powerful and ideally suited to detecting epistasis between the 30 genes. The approach is also ideally suited to testing interaction between groups of genes: the neurobiological pathways to which the candidate genes belong will be used to specify intra- and inter-pathway interactions. Importantly, the large sample size available to us will ensure that the screen for epistasis is both comprehensive and rigorous (i.e. controlling for multiple testing).

#### General Statistical Issues: Population Stratification & Selection Bias

##### Population stratification

We will control for potential false positive genetic effects caused by population stratification by using the panel of AIMs to estimate ancestral proportions by Bayesian cluster analysis implemented in the programs STRUCTURE[111,112] and L-POP.[113] This marker set has previously been shown to be sufficient in distinguishing ancestry of in an American sample accurately[114,115] and has been used to adjust for population stratification in a study of genotype by child maltreatment interaction in depression.[65,66] and in the 2004 Florida Hurricane study.[54]

##### Selection bias and missing data

Selection bias in a measure of exposure-disease association will result when the probability of being included in the study population depends jointly on disease status and exposure after properly controlling for confounders. Selection bias is a missing data problem; participants who opt out of the selection process (i.e. decline to be interviewed) will be missing from the final sample used in data analysis as will participants with incomplete data on analytic variables. Ultimately, we want the parameter estimates in our final models to be unbiased and, therefore, represent the population from whom our sample was drawn. The potential for selection bias exists if inclusion in the final sample depends jointly on genotype and PTSD. In most PTSD candidate gene association studies, such bias cannot be evaluated because the underlying population from which the cases and controls are drawn is not defined. A major strength of the current study is that cases and controls are nested within a larger cohort. We minimize the potential for selection bias in this study by having a clear definition of the underlying population, using explicit criteria for case-control selection, and selecting cases and controls from the same underlying population. We will also be able to systematically examine how the women who consent to diagnostic interviews differ both from those who do not consent and from the larger cohort as a whole on a large number of potential variables. We will then use the inverse probability weighting method to assess and adjust for selection bias in our analytic models. [116-118]

## Discussion

PTSD is a leading public health issue for American women. At this writing, this study will be the largest PTSD candidate gene study conducted to date and the only study in an all-female sample. Cases and controls will be carefully matched in terms of trauma history and, given 15 years of data on the cohort, we have the opportunity to consider a wide-range of confounders. Additionally, rather than examining only a single polymorphism per gene, we propose to comprehensively assay all common genetic variation in our candidate genes. This will provide a more complete assessment of the association between common variation within a gene and the development of PTSD than any work performed previously. Finally, our large sample size will enable us to move beyond detection of gene-disorder associations to dissection of the complex gene-trauma and gene-gene interactions underlying PTSD vulnerability. Taken together, these findings will set the groundwork for genomic studies aimed at verifying the functional significance of susceptibility haplotypes, clarifying their role in the etiology of PTSD, and examining their relevance to the development of new pharmacological treatments.

New treatments for PTSD are needed. About 30–50% of PTSD patients do not respond well to sertraline and paroxetine, the only medications currently approved by the FDA to treat PTSD.[119,120] Moreover, there is growing interest in acute pharmacological interventions to prevent the development of PTSD.[68] The potential public health impact of such low-risk and effective pharmacological interventions could be profound. If proved safe and effective, they could be administered to large numbers of people in mass trauma situations (e.g. natural disasters) as a primary prevention strategy. The identification of genetic variants that mediate susceptibility to PTSD will provide further clues to the pathophysiology of the disorder. That, in turn should facilitate the search for newer more effective pharmacological agents for PTSD treatment and prevention. Finally, the identification of PTSD risk genetic variants will improve the ability to identify high risk trauma exposed individuals and, therefore, target early intervention to those most in need.

### Potential limitations

There are four major limitations to this study. First, PTSD is highly comorbid; significant associations may not be specific to PTSD. As we have noted previously, PTSD but not trauma exposure without PTSD is highly comorbid with other psychiatric disorders. Moreover, a substantial proportion of this comorbidity is explained by common genetic influences.[48-50,52] This fact has led to the suggestion that PTSD represents a generalized vulnerability to psychopathology following trauma.[42] Thus, it is to be expected that some of the genetic variants associated with increased risk of developing PTSD would also be associated with increased risk of other mental disorders. Rather than viewing PTSD comorbidity as a problem, we view identifying significant PTSD-gene association as a first step in disentangling the complex relations between PTSD and other psychiatric disorders. Future research will need to follow-up on significant PTSD-gene associations and clarify which are unique to PTSD and which may represent a broader underlying vulnerability to psychopathology.

Second, diagnoses are being made by lay interviewers not experienced clinicians. Diagnosis by clinician via a structured interview, such as the Clinician Administered PTSD Scale (CAPS[121,122]), conducted face-to-face is generally considered to be the gold-standard for PTSD diagnosis. We have chosen to use a lay-administered structured interviewed conducted via telephone to diagnose PTSD and major depression. This decision was based on four considerations. First, the instruments we have chosen to use for PTSD and major depression diagnoses have both been validated against clinician diagnoses. A validation study was conducted by Dr. Breslau[92] found high agreement between the telephone interview and independent clinical re-interviews conducted by two clinicians blind to respondents' initial PTSD diagnosis (sensitivity = 95.6%, specificity = 71.0%). Acceptable-to-good concordance between the CIDI major depression diagnoses and blind clinical diagnoses has also been established.[94] Second, given the geographic distribution of the sample, in-person interviews were out of the question. Research has supported the validity of phone interviews as compared to face-to-face interviews for PTSD[93] and for depression.[95,96] The third major consideration in our choice of diagnostic procedure was cost. We were quoted a cost of $300 per clinician-administered interview; the cost per lay-administered interview from SRBI is approximately $50 per interview. The use of clinician-administered interview would have mean greatly reducing our sample size. Fourth, the most likely effect of the potential misclassification of cases and controls due to the use of lay-interviewers will be to reduce power and bias our results toward the null. Thus, any positive findings from this study are likely to be conservative estimates of PTSD-candidate gene associations.

Third, members of the NHSII are not representative of the general population of American women. Participants are not a random sample of US women, so the issue of generalizability to the general population must be considered. In particular, most NHSII participants of NHSII are Caucasian, middle to upper socioeconomic status (SES) and will be middle-aged (42–62 years of age) at the time of the current study. The homogeneity of the sample offers some advantages to our genetic analyses, e.g. population stratification. Moreover, evidence suggests genetic influences on some traits (e.g. IQ[123]) may magnified among more advantaged (higher SES) social groups. It is also important to note that the distribution of most risk factors in the NHSII is generally similar to the population at large; the frequencies of abuse reported in NHSII are similar to those in the National Violence Against Women Survey (54% and 52%, respectively, for childhood physical abuse; 17% and 18% for lifetime rape).[83,99] We will be unable, however, to generalize our findings to minority, poor, younger or older women and men. Moreover, "trauma exposure is a socially-patterned event.(p.234)"[124] Social context has been shown to moderate genetic effects;[123,125,126] the MAOA genotype-maltreatment interaction in predicting antisocial behavior was recently replicated Caucasian but not African-American males.[64] Social context is likely to be an important determinant of trauma exposure and PTSD at the population level.[124] Our analytic approach focuses on individual level determinants of PTSD among women in one fairly narrow social context. Replication in other epidemiologic samples from more heterogeneous social contexts will be required to determine whether positive findings from our study generalize and are meaningful at the population level.

Fourth, GWAS are state of the art; candidate gene studies are perceived to be outdated. We are enthusiastic about GWAS and several members of our research team are directly involved in such studies. However, we believe that, at this time, the candidate gene association study still offers the most efficient method for identifying genetic determinants of PTSD in women. As stated in their recent review of GWAS, Hirschorn & Daly argue "Before numerous expensive genome-wide association studies are attempted, we suggest pilot experiments should be used to test the merits of this approach" (p.105).[127] A GWAS of PTSD would be cost-prohibitive and would not provide as complete coverage of individual genes as can be accomplished in our candidate gene study. Thus, signals detected by a GWAS would still need to be followed up by fine-mapping of specific genes. Our approach will maximize coverage of common variation in specific genes that are strong candidates and will go further than any previous work toward providing answers about the role of these genes in the disorder. We would like to note that upon the completion of this project we will have trauma exposure, PTSD, and major depression data on 3,000 women with banked plasma DNA samples. Thus, it will be entirely feasible to conduct a whole genome association study on this cohort in the future if such studies prove to be economically feasible and scientifically justified.

### Conclusion and Future Directions

At the conclusion of this study, we will have PTSD screening data on over 60,000 women and PTSD diagnostic data on 3,000 women. The addition of trauma exposure and PTSD data to the NHSII cohort will provide an unparalleled resource for future investigations. Such investigations include the potential to efficiently conduct GWAS of PTSD and replication studies of positive PTSD-candidate gene associations found in this study. Moreover, chronic traumatic stress related to trauma and violence (even remote childhood exposure) is associated with lasting biological changes potentially important to the pathophysiology of many physical diseases among women, including cardiovascular and respiratory disease. [128-134] The inclusion of PTSD assessment data within the context of the established NHSII infrastructure designed to study the epidemiology of common disease will provide an unparalleled opportunity for the prospective examination of PTSD – disease associations. In particular, there will be the unique opportunity to study the effect of PTSD on risk of incident disease and to examine the underlying genetic and environmental mechanisms linking stress-related psychopathology to common physical disease outcomes.

## Abbreviations

PTSD: posttraumatic stress disorder; MD: major depression; SNP: single nucleotide polymorphism.

## Competing interests

The authors declare that they have no competing interests.

## Authors' contributions

KCK developed the background and design of the study and drafted the manuscript. ID, SMP, JRE, JWS, and RJW contributed to the background and design of the study. SMP developed the statistical approach and conducted the power calculations for the study. ID, SMP, JRE, JWS, and RJW revised the manuscript for important intellectual content. All authors approved the final manuscript.

## Pre-publication history

The pre-publication history for this paper can be accessed here:


